# Supplementary material for: Genetic profiling of Vietnamese population from large-scale genomic analysis of non-invasive prenatal testing data
Source: Sci Rep. 2020 Nov 5;10:19142. doi: 10.1038/s41598-020-76245-5 (PMC7644705; doi:10.1038/s41598-020-76245-5)
Supplement: Supplementary file 1 — Supplementary Information. [file 41598_2020_76245_MOESM1_ESM.pdf]

Genetic profiling of Vietnamese population from large-scale genomic analysis of non-invasive prenatal testing data

Ngoc Hieu Tran[1,7], Thanh Binh Vo[1,2], Van Thong Nguyen[3], Nhat-Thang Tran[4], Thu-Huong Nhat Trinh[5], Hong-Anh Thi Pham[1,2], Thi Hong Thuy Dao[1,2], Ngoc Mai Nguyen[1,2], Yen-Linh Thi Van[1,2], Vu Uyen Tran[1,2], Hoang Giang Vu[1,2], Quynh-Tram Nguyen Bui[1,2], Phuong-Anh Ngoc Vo[1,2], Huu Nguyen Nguyen[1,2], Quynh-Tho Thi Nguyen[2], Thanh-Thuy Thi Do[2], Nien Vinh Lam[6], Phuong Cao Thi Ngoc[2,8], Dinh Kiet Truong[2], Hoai-Nghia Nguyen[6,*], Hoa Giang[1,2,*], Minh-Duy Phan[1,9,*]

[1]Gene Solutions, Vietnam

[2]Medical Genetics Institute, Vietnam

[3]Hung Vuong hospital, Vietnam

[4]University Medical Center, Ho Chi Minh city, Vietnam

[5]Tu Du hospital, Vietnam

[6]University of Medicine and Pharmacy at Ho Chi Minh city, Vietnam

[7]David R. Cheriton School of Computer Science, University of Waterloo, Canada

[8]Division of Molecular Hematology, Lund Stem Cell Center, Lund University, Lund, Sweden

[9]School of Chemistry and Molecular Biosciences, University of Queensland, Brisbane, Australia

*Corresponding authors.

Hoa Giang (gianghoa@gmail.com)

Hoai-Nghia Nguyen (nhnghia81@gmail.com)

Minh-Duy Phan (m.phan1@uq.edu.au); ORCID: 0000-0002-3426-1044

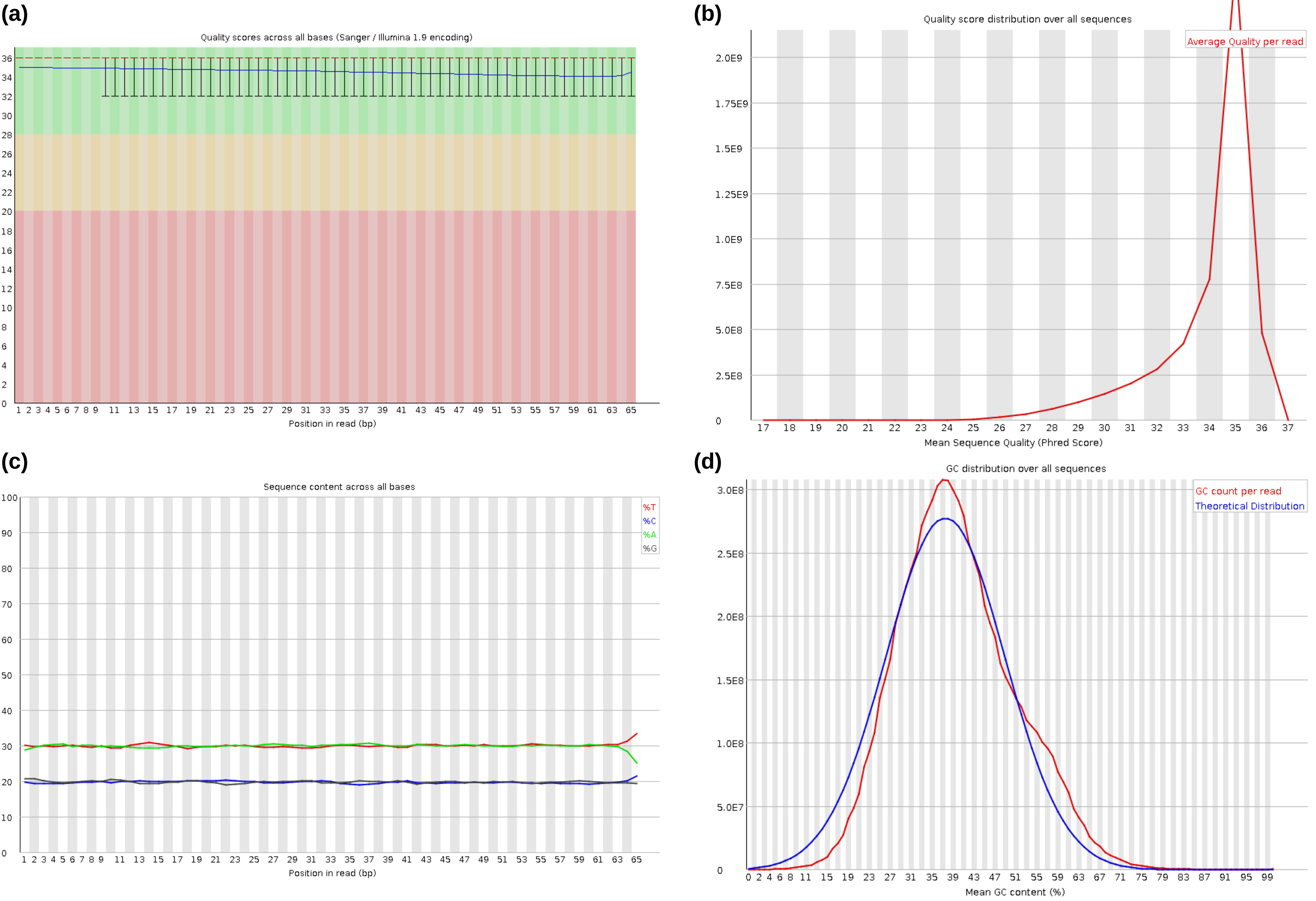

Figure S1. Quality control check on raw sequence data by FastQC. (a) Per base sequence quality. (b) Per sequence quality scores. (c) Per base sequence content. (d) Per sequence GC content. The quality control was performed on 1,000 samples randomly selected from our dataset

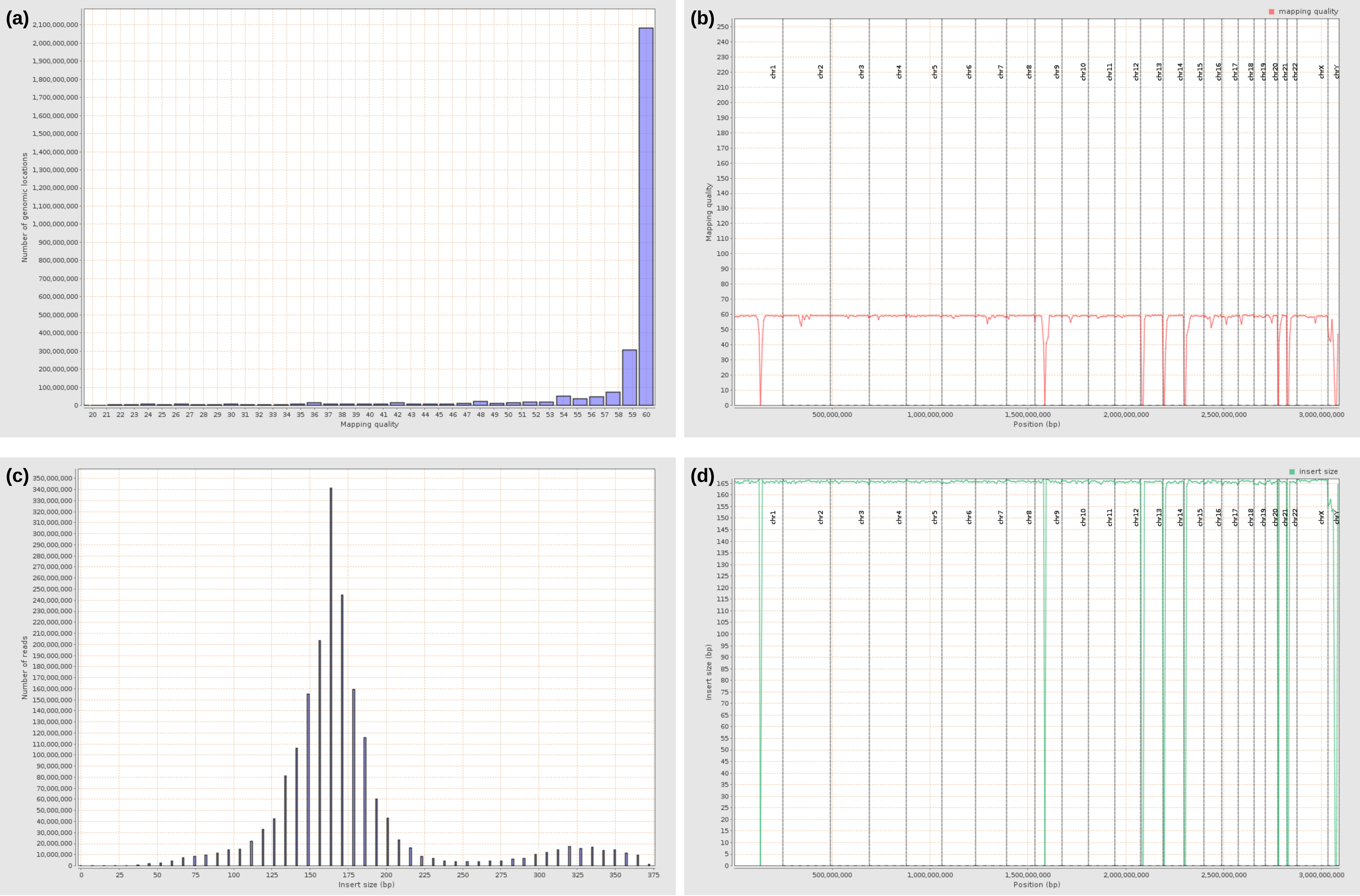

Figure S2. Qualimap report for the quality control of alignment results. (a) Mapping quality histogram. (b) Mapping quality across reference. (c) Insert size histogram. (d) Insert size across reference. The quality control was performed on 1,000 samples randomly selected from our dataset.

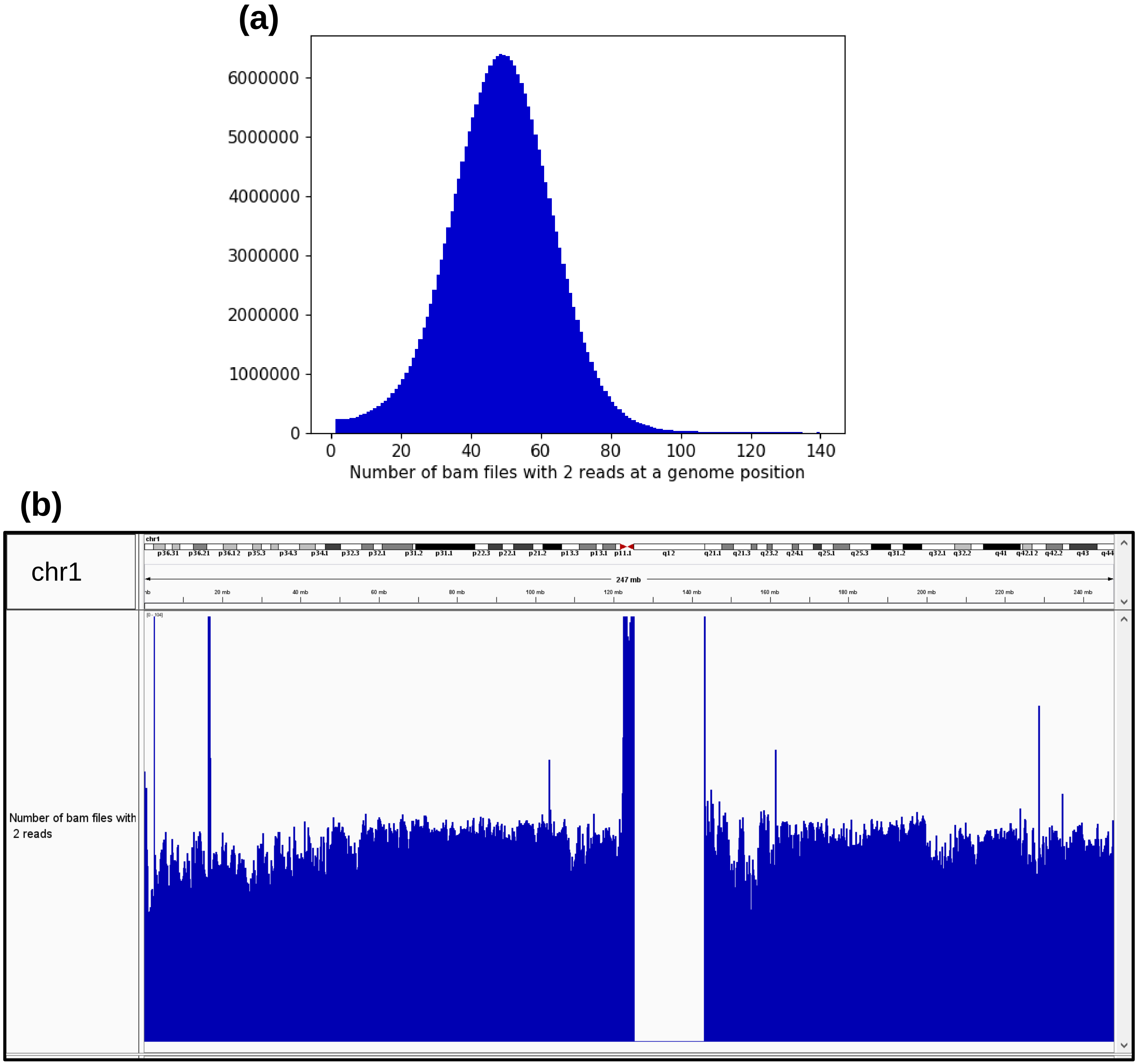

Figure S3. Distribution of the number of bam files that each contributed 2 reads at a genome position. (a) Summary histogram over all genome positions (y-axis shows the number of positions). (b) Distribution along chromosome 1.

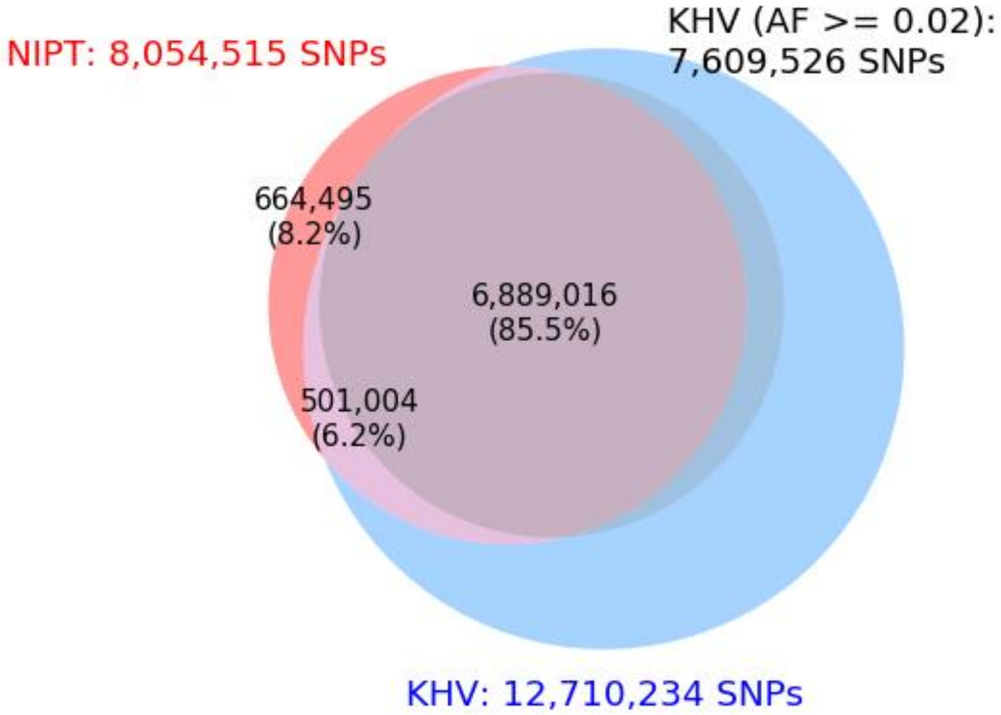

Figure S4. Venn diagram comparison between the NIPT call set, the KHV call set and its subset with allele frequency of at least 2%. The percentages were calculated with respect to the NIPT call set.

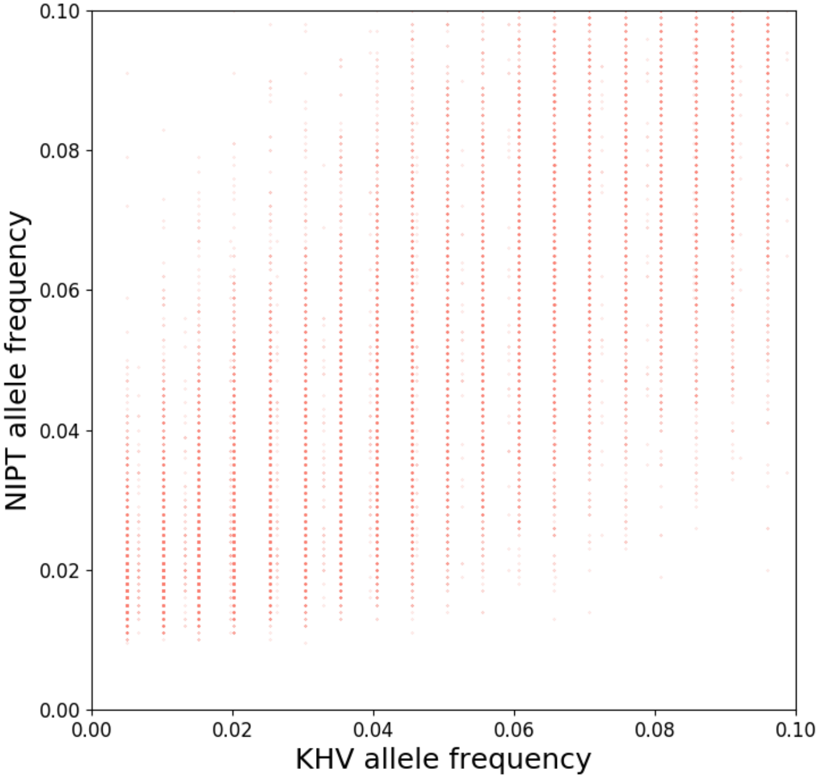

Figure S5. NIPT estimation of allele frequency showed better resolution than KHV's thanks to larger sample size.

Table S1. Pathogenic variants identified from the NIPT call set and their allele frequencies in the KHV population

Variant information					ClinVar annotations			Allele frequency		
chr	position	dbSNP	Ref	Alt	ID	Gene	Conditions	NIPT	KHV	Le *et al.* [11]
chr18	57571588	rs2272783	A	G	562	FECH	Erythropoietic protoporphyria	28.10%	25.76%	22.20%
chr13	20189473	rs72474224	C	T	17023	GJB2	Nonsyndromic hearing loss and deafness	13.40%	8.59%	9.64%
chr13	72835359	rs17089782	G	A	217689	PIBF1	Joubert syndrome	6.80%	4.55%	6.33%
chr6	26090951	rs1799945	C	G	10	HFE	Hemochromatosis type 1	5.10%	4.55%	3.82%
chr2	31529325	rs9332964	C	T	3351	SRD5A2	5-alpha reductase deficiency	2.90%	not found	1.36%

Table S2. ClinVar annotations of SNPs identified on gene GJB2.

Variant information					ClinVar annotations				Allele frequency		
chr	position	dbSNP	Ref	Alt	ID	Gene	Conditions	Interpretation	NIPT	gnomAD EAS	gnomAD
chr13	20188817	rs3751385	A	G	36277	GJB2	Nonsyndromic hearing loss, recessive	Benign	73.20%	55.56%	74.86%
chr13	20189503	rs2274084	C	T	36279	GJB2	Nonsyndromic hearing loss, recessive	Benign	15.50%	27.80%	5.04%
chr13	20189473	rs72474224	C	T	17023	GJB2	Nonsyndromic hearing loss, recessive	Pathogenic	13.40%	8.35%	0.76%
chr13	20189241	rs2274083	T	C	44739	GJB2	Nonsyndromic hearing loss, recessive	Benign	8.90%	18.81%	1.46%
chr13	20188974	rs76838169	A	G	44762	GJB2	Nonsyndromic hearing loss, recessive	Benign	3.30%	5.91%	0.42%
